# The COVID-19 Crisis: Skills That Are Paramount to Build into Nursing Programs for Future Global Health Crisis

**DOI:** 10.3390/ijerph17186532

**Published:** 2020-09-08

**Authors:** Teresa Peiró, Laura Lorente, María Vera

**Affiliations:** 1 Departamento de Enfermería, Universidad de Valencia, 46010 Valencia, Spain; 2 Institut d’Investigació en Psicologia del RRHH, del Desenvolupament Organitzacional i de la Qualitat de Vida Laboral (IDOCAL), Universidad de Valencia, 46010 Valencia, Spain; laura.lorente-prieto@uv.es; 3 Departamento de Educación y Psicología Social, Universidad Pablo de Olavide, 41013 Sevilla, Spain; mverper1@upo.es

**Keywords:** nurse, degree, training, pandemics, occupational stress, coping, transversal competencies, skills

## Abstract

The COVID-19 pandemic started at the end of 2019 and can be considered one of the most difficult health crises of the past century. It has had a devastating effect around the world, not only for public health, but also for the economy, labor market, and other facets of individual and societal life. Health systems have been put under high strain, and health professionals have experienced unusual and stressful work circumstances. With the aim of drawing lessons for nursing education, the present study analyzed, during the weeks of peak infection in Spain, the stress experience and coping strategies of a sample of 403 nurses from the Spanish health system. Specifically, we analyzed how tenure, stress appraisal, problem-focused coping, and support-seeking coping predicted nurses’ awareness of their education needs, both in terms of technical-professional knowledge and skills and transversal skills. Structural equation modeling analysis revealed that more tenure (years of experience) was related to lower stress appraisal (workload, −0.12, *p* < 0.05; insufficient preparation, −0.33, *p* < 0.001; and fear of contagion −0.36, *p* < 0.001) and more problem focused coping (PFC) strategies were related to higher awareness of professional (0.18, *p* < 0.01) and transversal educational needs (0.17, *p* < 0.01) while support seeking strategies just related to transversal training needs (0.10, *p* < 0.05). Moreover, the participants provided valuable input about specific contents to be considered in future nursing education programs. Implications for redesigning the nursing degree curriculum are analyzed in the discussion section.

## 1. Introduction

COVID-19 has been characterized as the most difficult health crisis and greatest challenge since World War II, and it has a global reach. In December 2019, an outbreak of a novel pneumonia appeared in Wuhan (Hubei, China), and since then it has spread all over the world, which has led to lockdown measures in many countries. In a short period of time, the pandemic emerged and grew in every continent, and the peaks moved from East to West and severely strained the health systems of most countries. In Spain, the ‘state of alarm’ caused by the COVID-19 outbreak was declared on the 14th of March 2020, and at that point most of the medical centers were already saturated. It is not just a health crisis because it has also important social, economic, and labor market impacts [1].

One of the most important features of this crisis is that it reflects “uncharted territory” in its biological, medical, therapeutic, and preventive facets. This and other facts have often led to the use of the metaphor of a war to describe the situation and its effects on the populations that have been subjected to lockdown [2,3].

COVID-19 has triggered tremendous public health challenges in many countries, including Spain, which has been overwhelmed by the volume, scope, and severity of the cases, the lack of information about the virus and the disease, uncertainty about its treatment, and the lack of a vaccine to prevent it. All of this has created important threats and uncertainties, as it already happened in previous pandemics in different parts of the world [4]. In Spain during the months of April and May 2020, the period where we carried out the survey of this study there was overwhelming patient demands. The situation was worsened by a lack of appropriate infrastructures, equipment, and resources, and the urgent need to adapt management systems [5]. Staff have had to work under conditions of extreme stress, due to the many facets of their work, the working conditions, and their personal and family lives.

Nurses usually work on the front lines of care, and they have the most contact with patients [6]. In this crisis, they play an important role facing complex COVID-19 cases that require hospitalization, often with preexisting health vulnerabilities and complications or mortality [7]. Thus, they work under physical and emotional pressure, putting their lives at risk while fulfilling their duties [8], especially when the protective equipment and safety procedures are insufficient and the work context is somewhat disorganized and chaotic. In this overwhelming context, it is important to study nurses’ work experiences, their main sources of stress and their strategies to cope effectively. Moreover, based on previous pandemics, such as influenza, Yonge et al. pointed out that “there is little attention paid during basic nursing education to emergency response, and faculty members report feeling poorly prepared to teach students about this topic” [9] (p. 24) thus, they claim that research should address “how emergency management principles can be incorporated into nursing curriculum” [9] (p. 28). In the COVID pandemic context this question arose from the real situation where recent graduates were suddenly hired to work in hospitals.

Aiming to obtain inputs from nursing experience during the COVID-19 pandemic we ask nurses about what they consider relevant education needs for the nursing profession, so that we may draw from their lessons learned in these special and unexpected circumstances. In addition, considering a detailed analysis of the psychological correlates we aim to identify significant predictors of the individual nurse’s views on this important anticipatory coping [10]. This coping strategy aims to prepare future nurses to cope professionally with the pandemic demands. Specifically, we first aim to identify the individual views about the education provided in nursing studies related to infectious disease outbreaks, in terms of both professional knowledge and transversal soft skills, such as communication with patients and management uncertainty under time pressure. Second, we also aim to identify relevant personal and work experience variables during the COVID-19 crisis to identify their predictor role on the views about nurses’ education needs. Thus, the research question is: during the COVID-19 pandemic, what relevant experiential antecedents at work influence nurses’ awareness of professional education needs related to pandemics and mass causality emergencies (MCE). We also aim to clarify how these experiences unfold to influence their views on those needs.

### 1.1. Nurses’ Work Stress during the Peak of the COVID-19 Pandemic: The Role of Work Experience

The peak period of the COVID-19 pandemic in Spain occurred in March, April, and May 2020. It was a period of heavy strain for the health system. However, the needs and concerns of nurses and other health professionals in these types of crises have not been fully described in the literature [11]. During the COVID-19 pandemic, the work context suddenly changed dramatically, and professionals had to work with insufficient resources, ward and intensive care unit (ICU) shortages of beds and equipment, and an overload of infected patients. All of this led to the reorganization of services and processes, with limited effective coordination and management, a lack of personal protective equipment, and uncertainty about infected cases due to a lack of tests to identify them because some could be asymptomatic. In these situations, regular stressors became acute and exacerbated.

Several theories of stress shed light on these stressful conditions. More specifically, the job demands–resources model [12,13] states that when at work demands are higher than the resources available to effectively fulfill them, the distress increases. Thus, this model clearly predicts higher levels of stress in these circumstances. The demands are extremely high in several ways. This includes the quantitative work overload due to the large number of patients [14]. The qualitative overload was also high, due to a lack of knowledge and high uncertainty about the virus and routes of infection, treatments, and the severity of the effects and consequences [15]. There were severe emotional demands due to the fear of infection and concern about being a potential carrier of the virus to their own family [16], as well as demands in dealing with people who are dying [14,15,17]. The stress was exacerbated by a shortage of resources. This was apparent and it was identified in a survey to health professionals (*N* = 2495) carried over by the foundation/institute for the sanitary assistance improvement (IMAS) [18]. First, there was a shortage of external resources, in terms of facilities [19], equipment, and staff [20] to provide decent service. There was also a shortage of other external resources to protect the health professionals working in health services. Second, personal resources were appraised as insufficient by some professionals, such as limited knowledge and qualifications for the unexpected and excessive uncertainties about the disease and treatment [9,21]. There were also changes in the work characteristics, with staff moving from their own service to respond to the new demands and job requirements [18]. Of course, occupational experience and tenure represented an important asset for health care professionals. First, professional experience is related to knowledge, skills, and competencies that are helpful when dealing with uncertain situations and problems. Second, the longer the experience as a health professional, the greater the possibility of having faced previous infection epidemics and crises, such as the SARS epidemic in 2003 or influenza A H5N1 in 2008–2009. This experience is a resource for managing stress in these uncertain circumstances. We assume that the years of practice will have a negative relationship with the level of stress appraisal in the special circumstances of this pandemic [7,8].

The conservation of resources (COR) theory [22] provides a theoretical base to study nurses’ stressful experience. According to this theory, “stress occurs (a) when central or key resources are threatened with loss, (b) when central or key resources are lost, or (c) when there is a failure to gain central or key resources following significant effort” [22] (p. 103). Moreover, one important principle of this theory states that “when people’s resources are outstretched or exhausted, they enter a defensive mode to preserve the self which is often defensive, aggressive, and may become irrational” [22] (p. 106). During the pandemic, people experienced an important loss of key resources such as employment [23]. Health professionals also experienced threats to personal (e.g., the lack of school for their children) and professional resources (e.g., the regular type of work and the normal levels of safety) [18]. Even more importantly, they experienced fear due to the risk of important losses. Most stressful experiences occur when professionals are confronted with outbreaks of lethal infectious diseases and are afraid of getting infected (or infecting others) [16,24]. Within this context, “nurses may experience high levels of psychological and emotional stress because health care systems will vary in their capacity to operate during large-scale public health emergencies” [11] (p. 347). At the same time, the chronic and overly taxing situation drives the nurses’ body, mind, and spirit into a state of tension, anxiety, and fatigue, and when these experiences cannot be alleviated over time, burnout may become a health problem [14]. Therefore, research is needed to study nurses’ stress in this complex, unexpected, and threatening situation. Recent empirical evidence has shown that nurses who supported COVID-19 treatment in Wuhan experienced tremendous pressure. Their stress was higher when they worried about their families, spent a higher number of hours per week working, and experienced higher anxiety while working [14]. Koh et al. [25], in a comprehensive systematic review of healthcare workers’ perceptions of risk in previous pandemics, found that health professionals experienced stress due to their personal exposure to health risks and the threat this represented for their loved ones. They also reported stigmatization by their neighbors as a social source of stress because of their exposure to respiratory infectious diseases. These authors found that “organizational implementation of infection control measures, avoidance of patients and complying with personal protective equipment were identified as risk-mitigating strategies” [25] (p. 403).

In this study, we aim to assess the demands and resources of nurses during the COVID-19 pandemic in Spain, and identify the role of tenure as an important resource that is negatively related to stress experiences. According to the COR theory, tenure and seniority are considered “condition resources” [22]. Thus, years of experience, as an indicator of tenure, may be considered a significant antecedent of stress due to high demands and low personal resources. With this rationale in mind, we formulated the following:

**Hypothesis** **1.** *Years of work experience will be negatively and significantly related to workload, insufficient preparation, and fear of contagion, as appraised by nurses.*

### 1.2. Nurses’ Coping with Stress during the Peak Period of the COVID-19 Pandemic

The response to the COVID-19 outbreak during the peak period of the pandemic required great effort from nurses to cope with these stressful experiences. Coping is a crucial element in the stress process because it plays a relevant role in the development of its consequences. Lazarus and Folkman [26] defined coping as cognitive and behavioral efforts to manage specific external and/or internal demands appraised as taxing or as exceeding the person’s resources [26] (p. 141). As Rodríguez et al. [10] state, “coping with stress is a fundamental element that determines the capacity to reduce and control the damage and costs that stress can provoke” [10] (p. 87). Literature has distinguished several types of coping. Dewe [27] classified the different types into two simple groups: problem-focused coping (PFC) and palliative or emotion-focused coping. PFC refers to a response of attempting to eliminate a perceived threat. Indeed, the most effective prevention strategy for work stress-related outcomes is to reduce sources of stress at work. Palliative or emotional coping aims to lessen the emotional discomfort triggered by the situation. Greenglass, Fiksenbaum, and Burke pointed out that palliative-focused coping often produces inconsistent results, and they recommend isolating social-support seeking from other types of emotional coping [28]. A meta-analysis by Viswesvaran, Sanchez, and Fisher [29] indicated the important role of social support in coping with stress. In the context of this research, it is worth examining the potential role of support-seeking coping (SSC). Several authors have pointed out that, instead of viewing some coping strategies as superior to others, it is important to adapt them to the particular demands and context [30].

This study focuses on direct action and social support seeking as two types of adaptive coping. We assume, especially in this highly threatening situation, that the skills to cope with the problems are important resources of nurses. In fact, nursing students faced with high perceived demands more often used coping behaviors that focused on the problem itself than those that focused on emotion [31]. However, the lack of personal resources reduced the motivation to initiate problem-focused coping strategies [32]. Thus, when stress resulted from the lack of capacity to deal with the demands, direct problem-focused coping (a stress-management strategy based on qualification to suppress the stressor) was less likely to occur, and other strategies such as support-seeking coping were expected to play a more significant role. With the previous rationale in mind, we establish the following hypotheses:

**Hypothesis** **2a.** *The higher the workload and fear of contagion, the higher the enactment of problem-focused coping.*

**Hypothesis** **2b.** *The higher the insufficient preparation appraisal, the lower the enactment of problem-focused coping.*

**Hypothesis** **3.** *The higher the workload, the insufficient preparation, and the fear of contagion, the higher the enactment of support-seeking coping.*

### 1.3. Input for Nursing Education from Nurses’ Professional Experience

During the COVID-19 pandemic, nurses gained valuable learning and priceless information, knowledge, and practice about how to deal effectively with many burdens of the health crisis [3], but they needed additional information and training. Mo et al. [14], referring to the current COVID-19 crisis, highlighted the need to improve the knowledge and skills of the nursing staff and their response to the epidemic. Choi et al. [7] claimed that staff should be provided with education about “current COVID-19 issues and risks specific to their practice area (e.g., pediatrics, maternal-infant health, nursing homes, schools, places of worship)” [7] (p. 5). Wu et al. [33] pointed out that nurses who received COVID-19 epidemic training had better mental health levels than those who did not, and Shah et al. [34] described a resilience training program for medical practitioners that was developed during the influenza pandemic. They showed that, after the session, 76% of the participants felt better able to cope. This estimate was significantly higher than the proportion that felt prepared to deal confidently with the pandemic before the session (35%) [35].

The education of nursing students has also been considered. Wilkinson and Matzo [21] pointed out the educational needs of nurses to effectively respond in mass causality emergencies (MCE). These authors stated that “preparing the health care workforce to respond appropriately and effectively requires nursing education programs to take on the important role of training nursing students in preparation for and in response to large-scale disasters and MCEs” [21] (p. 72). Other authors noted that “there is little attention paid during basic nursing education to emergency response, and faculty members report feeling poorly prepared to teach students about this topic” [9] (p. 24). They recommend “having nursing students undergo mock exercises to assess their level of understanding” [9] (p. 28) of crisis management.

In the past two decades, the services sciences have emphasized competency-based curricula for the education of professionals following the T-shaped professionals’ model of education. Successful services sector employees must possess a depth of understanding about their professional field (the vertical part of the T), and they must also have a fundamental understanding of the other disciplines they collaborate with (the horizontal part of the T) [36] and “stronger communication and teamwork abilities than ever before in order to succeed in complex, global organizations and networks” [37] (p. 543). Taking these vertical (technical and professional) and horizontal (interdisciplinary and transversal) parts into account, we aimed to identify the nurses’ awareness of education needs related to pandemics and crises for nursing students. Awareness of education needs may become clearer when nurses cope with stressful demands and situations. Therefore, the need for competent professional preparation will also become more salient. In a similar way, when trying to cope with a stressful problem by looking for support from other colleagues, the importance of professional resources obtained through education may become more evident. In line with this rationale, we formulate the following hypotheses:

**Hypothesis** **4.** *The higher the problem-focused coping, the higher the perceived need for education in both professional and transversal knowledge and skills.*

**Hypothesis** **5.** *The higher the support-seeking coping, the higher the perceived need for education in both professional and transversal knowledge and skills.*

## 2. Method

### 2.1. Study Design and Procedure

A cross-sectional study was designed to obtain information through an online survey, using LimeSurvey [38] that could be answered by computer, tablet, mobile phone, or any other electronic device. The study proposal was reviewed and approved by the Human research Ethics Committee of the University of Valencia (#1593679710463_7or6_1057_1281168_PDFa1), and the study was conducted according to the principles of the Declaration of Helsinki. The introduction to the questionnaire included the institutional identity of the researchers, the time estimated to answer, a short explanation about the objective of the study, and an invitation to participate. All participants gave informed consent for their participation and signed the data protection declaration before the start of the survey. Confidentiality and anonymity of their answers were assured. Nurses who participated in our study were contacted through several social media platforms and filled out the questionnaire during the period between 2 April and 31 May. This period falls within the ‘state of alarm’, and it includes the maximum peaks of contagion and deaths due to COVID-19 in Spain.

### 2.2. Variables and Measurement Tools

The survey included a total of 93 questions. One question asked about the years of experience practicing the nursing profession, which is considered an indicator of tenure.

Stressors were assessed with the Spanish validation [39] of the nursing stress scale by Gray-Toft and Anderson [40]. The factors studied are: (1) Work overload, which assessed stressful situations due to nurses’ workload, staffing and scheduling problems, and inadequate time to complete nursing tasks and support patients emotionally. It was measured with four items (e.g., ‘Not enough staff to adequately cover the work demands of the unit’); (2) Insufficient preparation for dealing with work demands. This factor includes a lack of preparation to deal with emotional needs of patients or having insufficient information to deal with task demands. It is measured with four items (e.g., ‘I do not feel well prepared to help with the emotional needs of a patient’); (3) Fear of contagion, which was designed purposely for this study. It includes three items to assess the fear of being infected or infecting others (e.g., ‘I have been afraid of improperly using protective equipment’). All items were rated on a six-point Likert scale ranging from 0 (never) to 5 (always).

Coping strategies were measured using the scales of the brief Coping Orientation to Problems Experienced (COPE) [41] using the items of the Spanish validation [42,43]. Problem-focused coping (PFC) includes 4 items on active and planning coping (e.g., ‘I have been taking action to try to make the situation better’) and support-seeking coping (SSC) includes 4 items focusing on instrumental and emotional support (e.g., ‘I have been trying to get advice or help from other people about what to do’). All items were measured with a six-point Likert scale ranging from 0 (never) to 5 (always).

Training needs were measured with an ad-hoc two-scale questionnaire designed to identify relevant education needs for a pandemic crisis that should be included or extended during nurses’ formal education. One scale focused on professional knowledge and skills (3 items: e.g., ‘Knowledge about professional protection in a pandemic crisis’) and the other scale focused on transversal knowledge and skills (3 items. e.g., ’Teamwork in mass crisis situations’). All items were answered with a five-point Likert scale ranging from 0 (nothing) to 4 (totally). In addition, an open question was asked for other important education contents that should be included in the nursing curriculum, related to pandemics (“Please mention any other contents that you consider important for the education of nursing students concerning pandemics”). The scales were formulated after several interviews with nurses and nursing teaching staff to ensure face and content validity, and both scales showed satisfactory reliability (0.79 each). Construct validity was tested with a confirmatory factor analysis (CFA) showing that the data corresponding to the three items from the professional training needs factor and those corresponding to the transversal training needs factor showed a good fit to the model (χ^2^ = 9.64, df = 6, *p* < 0.001; root mean square error of approximation (RMSEA) = 0.06; goodness-of-fit index (GFI) = 0.97; adjusted goodness-of-fit index (AGFI) = 0.92; incremental fit index (IFI) = 0.99).

### 2.3. Data Analyses

Descriptive analysis, internal consistencies (Cronbach’s alpha), and correlations among the variables under study were computed using the IBM-SPSS 26.0 program (IBM, Chicago, IL, USA). Structural equation modeling (SEM) was computed with AMOS 26.0 (IBM, Chicago, IL, USA) [44] to test the hypothesized model with the maximum likelihood estimation method. The input for each analysis was the covariance matrix of the items. The goodness-of-fit of the model was evaluated using absolute and relative indices. The absolute goodness-of-fit indices calculated were: (1) the chi-squared (χ^2^) goodness-of-fit statistic; (2) the root mean square error of approximation (RMSEA); (3) the goodness-of-fit index (GFI); and (4) the adjusted goodness-of-fit index (AGFI) [45]. Since the χ^2^ test is sensitive to the sample size, the calculation of relative goodness-of-fit indices is strongly recommended [46]. The following relative goodness-of-fit indices were calculated: (1) incremental fit index (IFI) and (2) comparative fit index (CFI) [47]. Since the distribution of the GFI and the AGFI is unknown, no statistical test or critical value is available [45]. As a rule of thumb, values near 0.08 for RMSEA indicate an acceptable model fit, and those smaller than 0.08 indicate a good model fit [48]. Relative fit index values greater than 0.90 indicate a good fit [49]. Finally, because data were all self-reported, there were concerns that the results might be influenced by common method variance. The danger is that at least some of the observed covariation between them may be due to the fact that they share the same method of measurement. Therefore, using IBM-AMOS 26.0, a Harman’s one-factor test was conducted [50].

**Figure 1:**
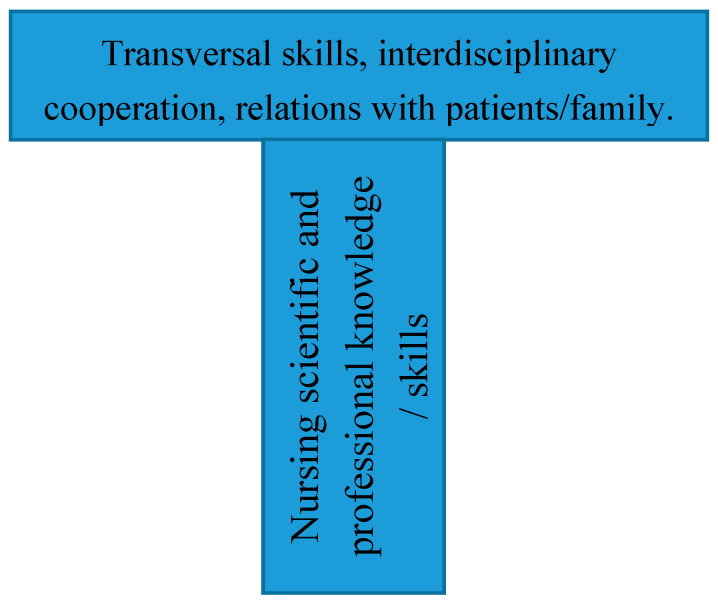
T model of the education in service profession.

In relation to the responses to the open question about input for the education of nurses, a content analysis was carried out to identify the main categories of education contents, both in terms of scientific and professional knowledge and skills and in terms of soft or transversal skills. The categorization was performed by two of the authors (one who was teaching nursing and the other work psychology), and the discrepancies were discussed and resolved by agreement. First the two broad blocks of contents were distinguished following the T model of the education in service sciences [36,37]: (1) contents related to the discipline and profession and (2) transversal skills and competencies relevant for the professional practice. Figure 1 shows the T model.

Then, the two experts agreed on a category framework for each of the blocks that was refined during the categorization process. Finally, they categorized the items corresponding to each category and revised the disagreements and found out by consensus the appropriate category. No software was used to carry over this categorization exercise.

## 3. Results

### 3.1. Participant Characteristics

In total, 403 nurses participated in the study out of the 779 who clicked the questionnaire (response rate, 51.74%). Of them, 93.5% were women and 6.5% men. Their mean age was 36.5 years old (SD = 10.5). They had considerable experience in their work, with more than 12 years on average (SD = 10.1). Regarding their workplace, 81.39% worked in hospitals, and the rest worked in other health centers throughout several Spanish provinces, mainly: Madrid 29.8%, Valencia 21.5%, Castellon 17.4%, and Barcelona 7.3%. Finally, 35.6% worked under a permanent contract, 47.7% were temporary, and 16.9% had another type of contract.

### 3.2. Descriptive Results

Means, standard deviations, Cronbach’s alphas, and correlations among the study variables are presented in Table 1. All the measures used present good reliability. Work tenure was significantly and negatively correlated with each of the stressors, showing that the higher the work experience, the lower the levels of stress appraisal. The correlations between stressors were all positive and significant because they identify several facets of occupational stress. In addition, workload and fear of contagion positively and significantly correlated with problem-focused coping (PFC; 0.17 and 0.16), indicating that the higher the nurses’ appraisal of these stressors, the more frequently they take actions to cope with them. On the other hand, insufficient preparation did not show a significant negative correlation with PFC or support-seeking coping (SSC). Finally, PFC significantly correlated with both education factors (0.16 each), and SSC significantly correlated (0.14) with the need for transversal-skills education. Results of Harman’s single factor test to detect common method variance (CMV) revealed a significant poor fit of the one-factor model (Delta χ^2^ = 387.82, *p* < 0.001), which means that CMV was not a serious problem in this study.

### 3.3. Hypothesis Testing

**Figure 2:**
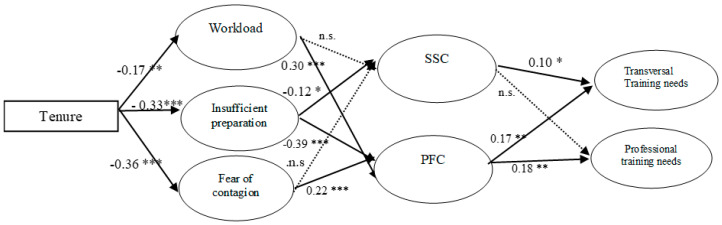
Research model and SEM results. * *p* < 0.05; ** *p* < 0.01; and *** *p* < 0.001.

The SEM computed to test our hypotheses showed that all the fit indices met the criteria (χ^2^ (388, *N* = 403) = 741.10; RMSEA = 0.048; GFI = 0.893; AGFI = 0.871; CFI = 0.925; and IFI = 0.925). Figure 2 shows the path coefficients. These results show that years of experience, or tenure, was negatively related to the appraisal of each stressor under study, fully confirming Hypothesis 1 (a negative relationship between years of work experience and workload, insufficient preparation, and fear of contagion). In turn, workload and fear of contagion were positively related to PFC, whereas insufficient preparation was negatively related to PFC. Thus, Hypotheses 2a (a positive relationship between workload and fear of contagion and the use of PFC) and 2b (a negative relationship between insufficient preparation appraisal and the use of PFC) were also confirmed. However, insufficient preparation was significantly and negatively related to SSC; thus, Hypothesis 3 (a positive relationship between workload, insufficient preparation, and fear of contagion SCC) was not confirmed, and an unexpected negative relationship between insufficient preparation and SSC was found. Hypothesis 4 (positive relationship between PFC and education needs for both professional and transversal knowledge and skills) was confirmed because PFC significantly predicted professional and transversal training needs. Finally, Hypothesis 5 (positive relationship between SSC and education needs for both professional and transversal knowledge and skills) was partially supported because SSC significantly and positively predicted transversal training needs, but not professional training needs.

In summary, tenure, or years of work experience, determined the appraisal of workload, insufficient preparation, and fear of contagion stress. In turn, these stressors were positively related to PFC, except in the case of insufficient preparation, which shows a negative relationship with PFC. In addition, the stressors did not have any impact on SSC, except in the case of insufficient preparation, which unexpectedly presents a negative relationship. Finally, as hypothesized, both types of coping behaviors significantly predicted education or training needs, although SSC only significantly predicted the level of transversal education needs.

### 3.4. Content Analysis of Qualitative Data on Education Topics

The qualitative analysis of the recommendations made by the nurses who participated in the study were also worthy of consideration. Seventy (17.4%) participants, out of 403, offered a total of 112 education contents (average, 1.6). The categories and frequencies of the inputs provided are presented in Table 2.

As Table 2 shows, 58.93% of the aforementioned contents refer to transversal soft skills (e.g., communication and relationship skills, team work organization and management, leadership, and teaching skills), and the rest to technical or professional skills. The main transversal skill mentioned refers to stress, coping, and emotion management (22 mentions), followed by communication and interpersonal relations (14 mentions). Regarding the scientific, technical, and professional knowledge and skills, the nurses emphasize basic training to work in important services of the hospital (22 mentions). They also refer to specific contents related to mass crises and pandemics (13 mentions).

## 4. Discussion

The present study analyzed and extracted lessons from professional nursing practice during the peak period of the COVID-19 pandemic in Spain that are relevant for nursing education. Our study aimed to identify the experiential antecedents that significantly predict nurses’ awareness of the need to extend both professional knowledge and transversal skills about pandemics in nursing education. Our results showed that tenure, defined as years of experience, is a protector against stress in every facet considered: work overload, insufficient preparation, and fear of infection. These three stressors, in turn, predicted PFC. However, overload and fear of contagion were positively related to PFC, insufficient preparation presented a negative correlation. However, stressors did not predict SSC, except in the case of insufficient preparation, which presented a negative relationship, contrary to our hypothesis. The results indicated that the higher the insufficient preparation, the lower the SSC. These results suggest that a lack of preparation blocks both PFC and SSC because the lack of important personal resources [12,13] decreases motivation and opportunities to effectively cope with the demands of the stressful situation. It is interesting to note that, for both types of coping, the lack of sufficient personal resources reduced coping actions designed to solve the problem, even when the solution could come from other more experienced colleagues. It is also noteworthy that stressful situations such as a high workload and fear of contagion did not induce higher levels of coping to obtain instrumental or emotional support. Future research should clarify the reasons for this. Possible explanations to be tested could be that overwhelming situations inhibit this type of behavior, or that other professionals who are also dealing with high volumes of demands are not available to fulfill social support functions. In general, our results support the hypotheses derived from the COR and JD-R theories. Other studies should also pay attention to another theoretical framework that also contributes to understanding high levels of stress: the effort–reward imbalance [51]. This theory states that stress also comes from the awareness that there is a negative imbalance between the contributions made and the recognition received. During the peak of the pandemic, people and the media conveyed gratitude and recognition to nurses, physicians, and other healthcare professionals as heroes (for instance, with applause every evening at 20:00 in all the Spanish cities during the lockdown). However, other messages were not as positive for recognition. For example, professionals complained that they were sent to the “battle front” with very limited protective clothing and equipment and important shortages in technology, staff, and other material resources to perform the required duties and demands [52]. Finally, those who had higher stress (and less experience), in their efforts to cope with the problems, are more aware of the need to include more professional and transversal knowledge and skills in nurses’ education. In addition, those with a higher frequency of support-seeking behaviors are also more aware of the need to educate nurses in skills required in these situations.

In sum, the results showed that, during the peak of the COVID-19 pandemic in Spain, nurses with higher stress and more active problem-focused coping became more aware of the need for future nurse education on public health crisis topics. As, Littleton-Kearney et al. [53] indicate, “nurses comprise a large percentage of the health care workforce, so that adequate educational preparation for nurses is essential” [53] (p. 103). They also point out that nurses remain unprepared to adequately respond to high-impact events. Thus, they emphasize that “education for nurses, built on the all hazards approach, provides the framework for college nursing program curricula, and for continuing education (CE) and just-in-time instruction” [53] (p. 103). The education of nurses as a service profession should take into account the T model emphasized by the service sciences [54] in order to better prepare nurses for interdisciplinary cooperation. According to this model, successful service employees must have in-depth preparation in their professional field (the vertical part of the T) and a good understanding of the other disciplines they collaborate with and transversal competences (the horizontal part of the T) [36]. The surveyed nurses’ emphasis on transversal and horizontal skills revealed that this facet was important to nursing professionals. However, it is somewhat surprising that no mention was made of digitalization. In this pandemic, it is apparent that proximity does not necessarily mean physical proximity, and tele-assistance, tele-diagnosis, and even tele-interventions can be effective and safe for curing and caring for patients, and also for providing social and emotional support from their families [17]. Therefore, one important area that needs more attention in the nursing curriculum is training in the necessary digital competencies for e-health and the different services and applications being developed and implemented in this field. The COVID-19 pandemic has increased the awareness of the need for digital technologies in healthcare. Physical distance does not prevent close interaction and presence when diagnosing or treating patients.

Some limitations need to be considered when interpreting the results. First, the data used in the model were gathered through a questionnaire at the same point in time (cross-sectional design). The Harman’s single-factor test showed that common method variance is not a deficiency in this dataset. However, the cross-sectional design, implying a lack of temporal sequence of the variables into the analysis, does not allow insights into causality. The sample due to online surveying does not match the gender or age distribution of the nurses’ population. In fact, when comparing the gender and age distribution of the population on the 31st of December 2019 [55] with the distribution of our sample we found out that women (population: 84, 2%; our sample 93, 5%) and young and middle-age professionals (<45 years old: population: 59, 9%; our sample: 77, 45%) were overrepresented in our sample. In addition, the questionnaire developed to identify the educational needs, although paying attention to both professional and transversal knowledge and skills, only covered a limited scope of relevant contents. Although we also included an open question to obtain complementary information on the relevant topics to be considered, future studies will have to develop more comprehensive questionnaires and more deeply reflect on the implications of public crises for the nurse curriculum. Insights obtained from the current study are useful for designing future studies aimed at improving nurses’ education related to pandemics or other health crises.

## 5. Conclusions

The present study identified significant inputs for nursing education related to pandemics and MCE, drawing from the experience of more than 400 nurses working mostly in hospitals during the peak COVID-19 lockdown period in Spain. During this period, an important saturation of patients occurred, with a significant shortage of material and personnel resources, both for protection and for appropriate clinical treatment. Our study has shown that professionals with higher stress tend to cope with stress by increasing problem-focused strategies. The exception was stress produced by insufficient preparation. The relationship between this lack of personal resources and both types of coping behaviors was negative.

Interestingly, the higher the frequency of coping behaviors, both problem-focused and support-seeking, the higher the awareness of educational needs in terms of knowledge and skills related to pandemics and other public health crises. The surveyed professionals provided important suggestions for the education of nursing students, although surprisingly there was no mention of e-health. There is a need for further analysis of the lessons learned from this unusual and exceptional experience, and their implications for nursing education.

## Figures and Tables

**Table 1:** Means, standard deviations, Cronbach’s alpha, and Pearson correlations (*N* = 403).

	M	SD	α	1	2	3	4	5	6	7	8
1. Tenure	12.8	10.1	-								
2. Work overload	2.20	0.81	0.81	−0.16 **	1						
3. Insufficient Preparation	2.08	0.87	0.74	−0.32 **	0.36 **	1					
4. Fear of contagion	3.08	1.08	0.65	−0.28 **	0.41 **	0.38 **	1				
5. PFC	3.39	0.94	0.88	0.01	0.17 **	−0.09	0.16 **	1			
6. SSC	2.97	1.00	0.82	−0.04	−0.09	−0.05	−0.01	0.28 **	1		
7. Professional Training needs	3.01	0.84	0.79	0.10 *	0.03	−0.07	0.02	0.16 **	0.07	1	
8. Transversal Training needs	3.10	0.88	0.79	0.03	0.09	−0.01	0.04	0.16 **	0.14 **	0.68 **	1

Note. * *p* < 0.05; ** *p* < 0.01. PFC: problem focused coping; and SSC: support-seeking coping.

**Table 2:** Content analysis of inputs provided by the nurses in the survey. Categories and frequencies of items mentioned in each category.

Blocks and Categories of the Inputs Provided by Participants in the Survey	Number of Mentions
***Block A: Scientific, technical, and professional knowledge and skills.***	46 41.07%
**Catastrophes (NBQ, epidemics) and drills**. Waste management, protection of vulnerable groups, prevention, etc.	13
**Basic training in important services**. Intensive Care Unit; Emergency; Surgery, Critical Care Resuscitation Unit; Palliative Care, etc.	22
**Specific techniques and technology use**. Use of technology, techniques (MARS), surgery rooms and surgical instruments, etc.	7
**Patients’ treatment and clinical aspects.**	3
**Research knowledge and skills.**	1
***Block B: Knowledge and skills about transversal soft skills.***	66 58.93%
**Professional deontology, attitudes, and other disciplines** (e.g., law)	7
**Stress, coping, and emotion management.** Control of stress, dealing with death and patients dying, etc.	22
**Self-protection and self-care**. Personal protection equipment, hygiene, etc.	5
**Communication and relationships** with colleagues and other professionals, with patients and relatives, empathy, interpersonal relations, etc.	14
**Teamwork, organization, and management and leadership.** Vertical upward and downward relations, resource management, team organization, etc.	7
**Teaching health to general public.** Teaching skills to teach general public, vulnerable groups, etc.	1
**Other.** Organizational and work inducement and socialization, etc.	10
**Total**	112
